# Regulation of L1 expression and retrotransposition by melatonin and its receptor: implications for cancer risk associated with light exposure at night

**DOI:** 10.1093/nar/gku503

**Published:** 2014-06-09

**Authors:** Dawn deHaro, Kristine J. Kines, Mark Sokolowski, Robert T. Dauchy, Vincent A. Streva, Steven M. Hill, John P. Hanifin, George C. Brainard, David E. Blask, Victoria P. Belancio

**Affiliations:** 1 Department of Structural and Cellular Biology, Tulane School of Medicine, Tulane Cancer Center, New Orleans, LA 70115, USA; 2 Tulane Center for Aging, New Orleans, LA 70112, USA; 3 Department of Neurology, Thomas Jefferson University, Jefferson Medical College, Philadelphia, PA 19107, USA

## Abstract

Expression of long interspersed element-1 (L1) is upregulated in many human malignancies. L1 can introduce genomic instability via insertional mutagenesis and DNA double-strand breaks, both of which may promote cancer. Light exposure at night, a recently recognized carcinogen, is associated with an increased risk of cancer in shift workers. We report that melatonin receptor 1 inhibits mobilization of L1 in cultured cells through downregulation of L1 mRNA and ORF1 protein. The addition of melatonin receptor antagonists abolishes the MT1 effect on retrotransposition in a dose-dependent manner. Furthermore, melatonin-rich, but not melatonin-poor, human blood collected at different times during the circadian cycle suppresses endogenous L1 mRNA during *in situ* perfusion of tissue-isolated xenografts of human cancer. Supplementation of human blood with exogenous melatonin or melatonin receptor antagonist during the *in situ* perfusion establishes a receptor-mediated action of melatonin on L1 expression. Combined tissue culture and *in vivo* data support that environmental light exposure of the host regulates expression of L1 elements in tumors. Our data imply that light-induced suppression of melatonin production in shift workers may increase L1-induced genomic instability in their genomes and suggest a possible connection between L1 activity and increased incidence of cancer associated with circadian disruption.

## INTRODUCTION

Long interspersed element-1 (L1) is a non-LTR (long terminal repeat) family of retroelements distributed throughout mammalian genomes (1,2). Both the germline and somatic human tissues support endogenous L1 expression (3) and *de novo* L1 retrotransposition (4,5). L1 mobilization requires transcription of the full-length L1 mRNA that can generate functional ORF1 and ORF2 proteins (ORF1p and ORF2p) (6) followed by the formation of an RNP (ribonucleoprotein) complex (7). As a result, a reduction in any one of the three components (ORF1p, ORF2p or mRNA) is expected to downregulate L1 mobilization.

**Figure 1.**
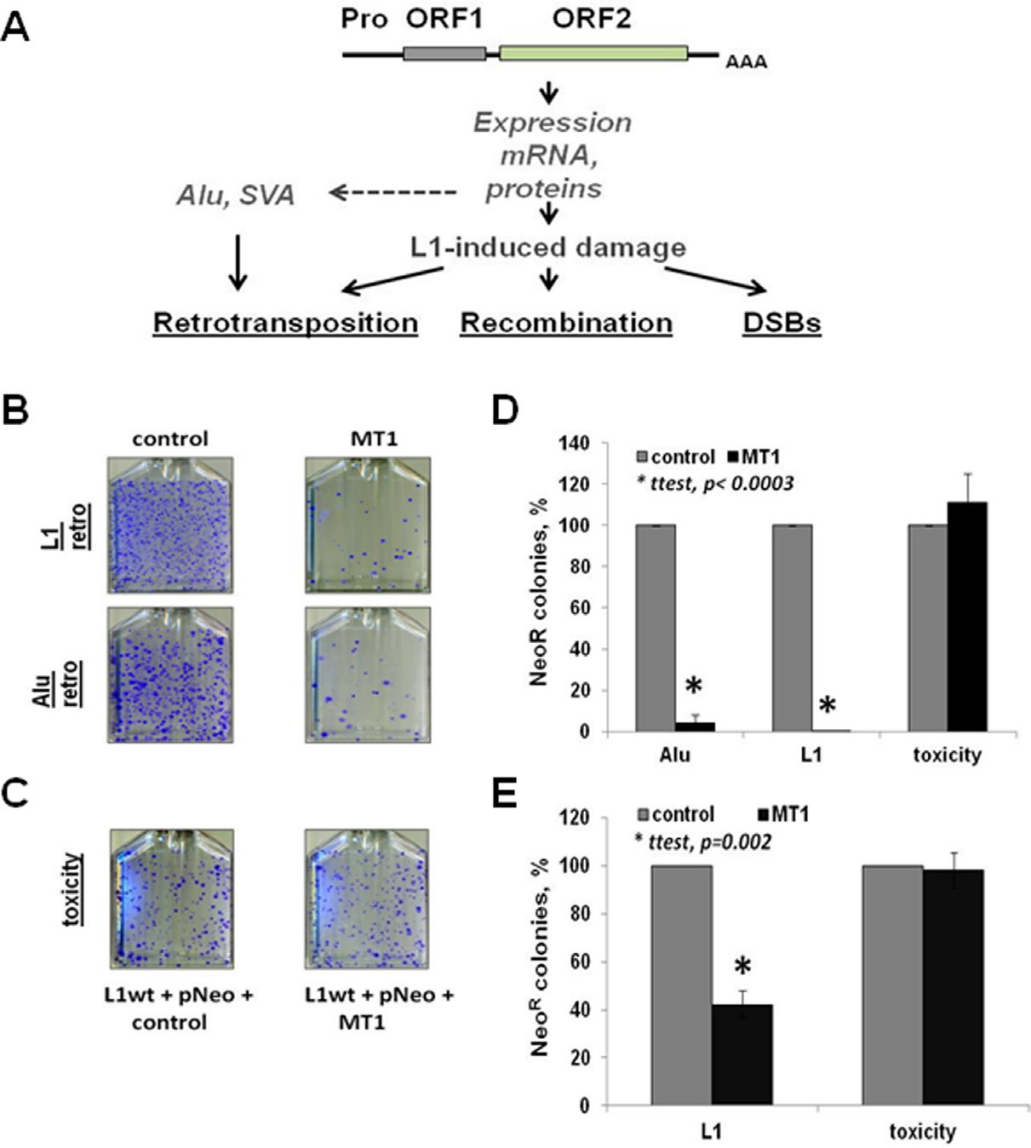
Melatonin receptor 1 inhibits L1 and Alu retrotransposition in cultured cancer cells. (**A**) Schematic of L1 and its damage. Pro is an internal polymerase II promoter present within the L1 5′UTR; ORF1 and ORF2 are L1-encoded proteins. AAA is a polyA tail. L1 expression produces L1 mRNA and proteins that can cause genomic instability through retrotransposition of L1, Alu and SVA (SINE-VNTR-Alu Element), as well as DNA DSBs, which depend on the endonuclease function of the L1 ORF2p. Accumulation of repetitive elements in the human genome to high copy number also leads to non-allelic recombination. (**B**) L1 and Alu retrotransposition (L1 retro and Alu retro) in HeLa cells transiently transfected with either control or MT1 expression plasmids (for experimental design, see Supplementary Figure S1). (**C**) L1 toxicity in the presence or absence of MT1 expression in HeLa cells. The toxicity assay is a colony formation assay using a neomycin expression vector cotransfected with an untagged L1 and control or MT1 expression plasmids to test the effect of L1 overexpression or any synergistic effect of L1 and MT1 overexpression on cell viability and colony formation (also see Supplementary Figure S2). (**D**) Quantitation of the effect of MT1 overexpression on L1 and Alu retrotransposition and L1 toxicity in HeLa cells. L1 and Alu retrotransposition potential (L1 or Alu) in the absence (gray bars) or presence (black bars) of MT1 overexpression. The same colors are used to represent toxicity from L1 and MT1. (**E**) Quantitation of the effect of MT1 overexpression on L1 retrotransposition and toxicity in PC3 cells. Error bars are standard deviation; asterisks indicate statistically significant differences by the *t*-test.

L1 ORF1p forms trimers that directly bind to L1 mRNA (8–10) and has a nucleic acid chaperone activity that is required for L1 integration (11). L1 ORF2p encodes three functional domains: endonuclease (EN), reverse transcriptase (RT) and a cysteine-rich domain (Cys) encoding a putative RNA-binding motif (6,12–15). Retrotransposition is initiated by the EN domain nicking the host DNA. The RT domain completes first strand cDNA synthesis, and cellular factors are likely involved in aiding the completion of L1 integration [reviewed in (16)]. The ORF2 EN domain is also responsible for generation of DNA double-strand breaks (DSBs) that are more abundant than L1 retrotransposition events (Figure 1A) (17). Additionally, due to their high genomic copy number, L1 (500 000 copies) and Alu (over 1 000 000 copies) are involved in non-allelic homeologous recombination, resulting in the loss or rearrangement of genetic information [reviewed in (18)].

Because L1 also provides the retrotransposition machinery for the parasitic Alu and SVA elements, it is the source of the entire mutagenic burden on the human genome imposed by active retrotransposons (6,19,20). All types of L1-induced genomic instability (*de novo* insertions, DSBs and non-allelic recombination, Figure 1A) can contribute to cancer origin or progression (5,16,21–24). L1 expression is upregulated in the majority of human cancers relative to normal tissues (3,7), often due to the loss of cellular functions known to suppress L1 expression (25,26). Recent literature strongly supports that L1 mutagenesis is a likely contributing factor in tumor origin and progression (5,22,23).

Numerous cellular pathways have been shown to control almost every step of the L1 replication cycle (18). These pathways range from transcription factors and RNA processing to DNA repair (27–31). However, very little is known about regulation of L1 expression *in vivo* because most L1 biology is studied in cultured cells lacking multiple regulatory mechanisms that are unique to living organisms. One such regulator is the circadian system that synchronizes the timing of biological processes ranging from metabolism to DNA damage response (32,33). While circadian oscillation is autonomous, it can be entrained to the periodicity of environmental light and darkness over a 24-h cycle. This entrainment translates into the synchronization of biological processes by the receptor-mediated action of nocturnal melatonin production. There are two major melatonin receptors in mammals, MT1 and MT2. The G-coupled melatonin receptor 1 (MT1) is widely expressed both within and outside the central nervous system (34,35). The MT1 receptor expression is increased to varying degrees in many cancers of epithelial origin, including breast, pancreatic and gallbladder cancers (36–39). Similar to melatonin production, MT1 receptor expression exhibits circadian variation (34,40). The pattern of melatonin production during the dark phase of the circadian cycle is the same in both nocturnal and diurnal animals (41), making rodents an effective model for studying circadian regulation relevant to human health. Light exposure at night suppresses melatonin production in humans (42) and rodents and is associated with an increased incidence of breast, prostate and other cancers in shift workers (43,44) and animal models (45,46). In 2007, the World Health Organization recognized nocturnal light exposure that entails circadian disruption as a probable carcinogen (47).

While the increased risk of cancer in shift workers is recognized, the molecular basis of this phenomenon is not known. We discovered that endogenous L1 expression is regulated by melatonin in a receptor-mediated manner *in vivo*. Combined with tissue culture data demonstrating MT1-induced suppression of L1 and Alu retrotransposition, this observation suggests that activation of L1-induced damage in the genomes of shift workers could be one of the causes underlying increased risk of cancer in this subpopulation.

## MATERIALS AND METHODS

### Cells

HeLa, PC3, immortalized human fibroblast (GM04429, Coriell) and NIH 3T3 cells were cultured in modified Eagle's Medium (MEM) with 10% fetal bovine serum (FBS), 100-mM sodium pyruvate, 1X non-essential amino acids and 200-mM l-glutamine and Dulbecco's modified Eagle's medium with 10% FBS, respectively.

### Plasmids

Plasmids CMV5′UTR L1Neo, ΔCMVL1Neo, and untagged CMV5′UTR L1 are from (6), untagged CMV5′UTR L1 ORF1stop is from (48), CMVΔ5′UTR L1Blast is from (49), codon optimized human L1 (co hL1) is from (50) codon-optimized human ORF1 expression plasmid (ORF1) is from (51), AluNeo is from (19,50), pCDNA, CMV Fluc, (Promega), 5′UTR Fluc are from (52), MT1 is from (53), Untagged ΔCMV L1 was generated by NotI and SalI digest of JM101 L1.3, followed by gel extraction and purification of the L1-containing fragment. The purified L1-containing sequence was cloned into pBluescriptII (SK) digested with NotI and SalI. ORF1 Ser codon-optimized sequence was synthesized to contain serine to alanine mutations at amino acid positions 12, 18, 25, 26, 27, 33, 50, 53, 106, 109, 119, 166, 208, 209, 281 and 290 of ORF1p. ORF1 Ub codon-optimized sequence was synthesized to contain lysine to alanine mutations at positions 223, 227, 229, 237, 243, 245, 272, 274, 285, 295, 300, 314, 318 and 337 of ORF1p; in addition, an EcoRV site at amino acid 179 was introduced without changing the amino acid composition. The synthesized sequences were cloned into pBudCE4.1 expression plasmid (Invitrogen) using HindIII and BamHI restriction sites.

### Prediction of putative phosphorylation and ubiquitination sites

NetPhos 2 and CKSAAP prediction programs were used to identify putative phosphorylation and ubiquitination sites in the ORF1p sequence (54,55).

### Retrotransposition assay

HeLa cells (500 000) were transfected 18–24 h after seeding by lipofectamine/Plus reagent (Invitrogen) with 0.4 µg of Neo-tagged L1 expression vector (6) and 0.1 µg of pCDNA or MT1 expression vectors. To control for MT1-associated toxicity, HeLa cells were transfected with 0.4 µg of pCDNA, 0.1 µg of pCDNA or MT1 expression vectors, and 0.8 µg of pIRES vector.

PC3 cells (1 × 10^6^) were transfected by lipofectamine/Plus reagent with 0.3 µg of Neo-tagged L1 expression vector and 0.06 µg of pCDNA or MT1 expression vectors 18–24 h after plating. For toxicity of L1 activity, melatonin receptor antagonist, melatonin receptor or their combination, L1 notag, or MT1 expression plasmids were used with 0.8 µg of pIRES (has Neo-resistance). Selection with 0.4 (HeLa) or 0.22 (PC3) mg/ml G418 was initiated 24 h after transfection and maintained for 2–3 weeks. Cells were treated with 10^−7^, 10^−6^, 10^−5^ M of melatonin receptor antagonist S20928 (a generous gift from the Institute Recherches de Servier, Cerbevoie, France) at the time of transfection cocktail removal, at the time of G418 addition 48 h after transfection and the following day.

### Western blot analysis

HeLa, polymerase chain reaction (PCR), NIH 3T3 and immortalized human fibroblasts were seeded and transfected with L1 and MT1 expression plasmids as described for RNA analysis below. Total protein lysates were harvested 24 h after transfection. For analysis of MT1 effect on wild-type and mutant ORF1p, cells were transfected with 3 and 0.5 μg of ORF1 and MT1 expression plasmids, respectively. Western blot analysis was preformed as previously described (51). Briefly, cells were harvested using Triton sodium dodecyl sulfate lysis buffer, TLB SDS (50-mM Tris, 150-mM NaCl, 10-mM ethylenediaminetetraacetic acid, 0.5% sodium dodecyl sulfate, Triton X 0.5%, pH = 7.2) supplemented with Halt Protease inhibitors and phosphatase inhibitors 2 and 3 (Sigma), for total protein harvest. Samples were sonicated three times for 10 s at 12 Watt RMS using 3-mm wide Microson ultrasonic cell disruptor XL2000 (Microson). Total lysates (10–40 μg) were analyzed by western blotting as described (51). Briefly, membranes were blocked for 1 h in 5% milk in phosphate buffered saline–Tween and incubated with hORF1 (custom polyclonal rabbit antibodies, epitope: TGNSKTQSASPPK) or hORF1 201 (custom polyclonal rabbit antibodies, epitope: QRTPQRYSSRRATP).

### Animals and tumor generation

Adult inbred male nude rats (Hsd:RH-*Foxn1^rnu^*) were implanted with PC3 human prostate cancer xenografts in a tissue-isolated manner as previously described (56,57).

### Tumor perfusion

PC3-derived tumors established in nude male rats (Hsd:RH-*Foxn1^rnu^*) were perfused with human blood as previously described (58). Human blood was collected from healthy adult male donors and preserved for perfusions as previously described (58). Animal handling and treatment was done according to the approved Institutional Animal Care and Use Committee (IACUC) protocol.

### RNA analysis

#### RT-PCR

Total RNA was extracted from tumor samples or cultured cells by Trizol (Invitrogen) and polyA selected as previously described (30). RNA was quantified and cDNA was synthesized using the Reverse Transcription System kit (Promega) according to manufacturer's protocol. For all of the samples in each experimental group, equal amounts of RNA were used in the reverse transcription reaction (Group 1 = 154 ng RNA, Group 2 = 169 ng RNA, Group 3 = 157 ng RNA). Reverse transcription was primed with random primers. As a negative control, additional reactions were performed without the RT. Polymerase chain reaction (PCR) was performed using 900 ng of cDNA from each reaction using GoTaq Hot Start mastermix (Promega) and 23 thermal cycles of 94°C for 30 s, 57°C for 30 s and 72°C for 10 s. The same cycling conditions were used for primer validation using DNA from rat or human cells. The 5′UTR region of human L1 was amplified using primers (5′-GCCAAGATGGCCGAATAGG-3′) and (5′-TGGCACTCCCTAGTGAGATGAA-3′) and a region spanning exon 3 and 4 of β-actin was amplified using primers (5′-ACCTTCTACAATGAGCTGCG-3′) and (5′-CCTGGATAGCAACGTACATGG-3′). Amplification products were fractionated by electrophoresis through 1.5% agarose and digital images recorded (Versa-Doc system; Bio-Rad).

Northern blot analysis was performed as previously described (28). In short, HeLa, PC3 and NIH 3T3 cells seeded at 2 × 10^6^ per T75 flask, were cotransfected 18–20 h after plating with 6 µg of the L1 expression plasmid and 0.5 µg of empty or MT1 expression plasmid (59), 12 µl of Plus and 24 µl of lipofectamine reagents (Invitrogen). Cells were harvested for total RNA extraction with Trizol (Invitrogen). Following polyA selection, RNA was fractionated by agarose–formaldehyde gel electrophoresis, transferred onto nitrocellulose membrane, cross-linked and analyzed with a strand-specific RNA probe complementary to the first 100 bp of the sense strand of L1 mRNA. To determine L1 mRNA stability, HeLa and NIH 3T3 cells transiently transfected with an L1 expression plasmid were treated 24 h posttransfection with 75 μg/ml of Actinomycin D for 2, 4 and 8 h. Collected RNA was analyzed by northern blot with a strand-specific RNA probe complementary to the first 100 bp of the L1 5′UTR as described (28).

### Luciferase assay

HeLa cells (100 000) per well (six-well plate) were transfected 18–24 h after plating with 0.2 µg of luciferase expression plasmids [luciferase expression is driven by either CMV (pGL3 pCMV, Promega) or L1 5′UTR promoters (52)], 0.02 µg of the MT1 expression plasmid and 0.02 µl of the pBIND plasmid (Mammalian two-hybrid system, Promega), which expresses Renilla luciferase. pGL3-basic (Promega) was used as a negative control. DNA was transfected using 1 µl of Plus and 1.5 µl of lipofectamine reagents (Invitrogen). The transfection cocktail was replaced with regular media 3 h after transfection. Cells were harvested for luciferase activity analysis 48 h after transfection. Protein extraction was performed according to the manufacturer's protocol (Promega). Protein concentrations were determined using standard BSA approach. The same amount of protein for each sample was analyzed using Promega dual-luciferase detection system.

## RESULTS

### Transient MT1 expression suppresses L1 and L1-driven Alu retrotransposition in cultured cells

We utilized L1 and L1-driven Alu retrotransposition assays in cultured cells (6,19) to investigate whether L1 activity may be regulated by the host's circadian system (Supplementary Figure S1). Specifically, we tested the effect of MT1 overexpression on L1 and Alu mobilization in two cell lines, HeLa and PC3, which represent cancers of an increased incidence in shift workers (60–62). Melatonin exhibits its anticancer effect primarily through its MT1 receptor because it is expressed in the central nervous system and most if not all peripheral tissues (34,35,40). MT1 receptor expression is also altered in many human cancers (36–39,63–65) and it is reported to associate with lower breast tumor stage and smaller tumor size (66). We performed transient transfections of a tagged-L1 expression plasmid [L1Neo, (6)] with either an empty plasmid or a construct expressing human MT1 receptor in HeLa cells (Figure 1B control and MT1, and Figure 1D gray and black bars, respectively). Transient expression of MT1 results in significant reduction in neomycin-resistant colonies (NeoR), which represent *de novo* L1 retrotransposition events in HeLa (Figure 1B and D) and PC3 cells (Figure 1E). Similarly, MT1 overexpression also suppresses L1-driven *de novo* Alu retrotransposition in cultured cells (Figure 1B and D). The activation of MT1 receptor under this experimental condition occurs through receptor overexpression and melatonin present in the serum.

The retrotransposition assay relies on colony formation that depends on cellular ability to support retrotransposition and colony growth. Any adverse effect of MT1 or L1 overexpression (17,67) on cell viability may cause a reduction in NeoR colonies. To test the specificity of MT1's effect on retrotransposition, we made use of a toxicity assay (Supplementary Figure S2) (17). The retrotransposition assay produces NeoR colonies only if a *de novo* L1 or Alu integration takes place (6,19). In contrast, the NeoR colony formation in the toxicity assay relies on random integration of a neomycin expression plasmid (pNeo, Supplementary Figure S2) into the cellular genome. Any difference in colony numbers between cells cotransfected with pNeo and untagged L1, MT1, or both expression vectors would indicate toxicity associated with their expression. No negative effect of MT1 overexpression on colony formation in HeLa or PC3 cells was detected supporting that MT1 expression specifically affects retrotransposition, (Figure 1C, toxicity, and Figure 1D and E toxicity, black bars).

### Melatonin receptor antagonists reverse MT1 suppression of L1 and Alu mobilization in a dose-dependent manner

**Figure 2.**
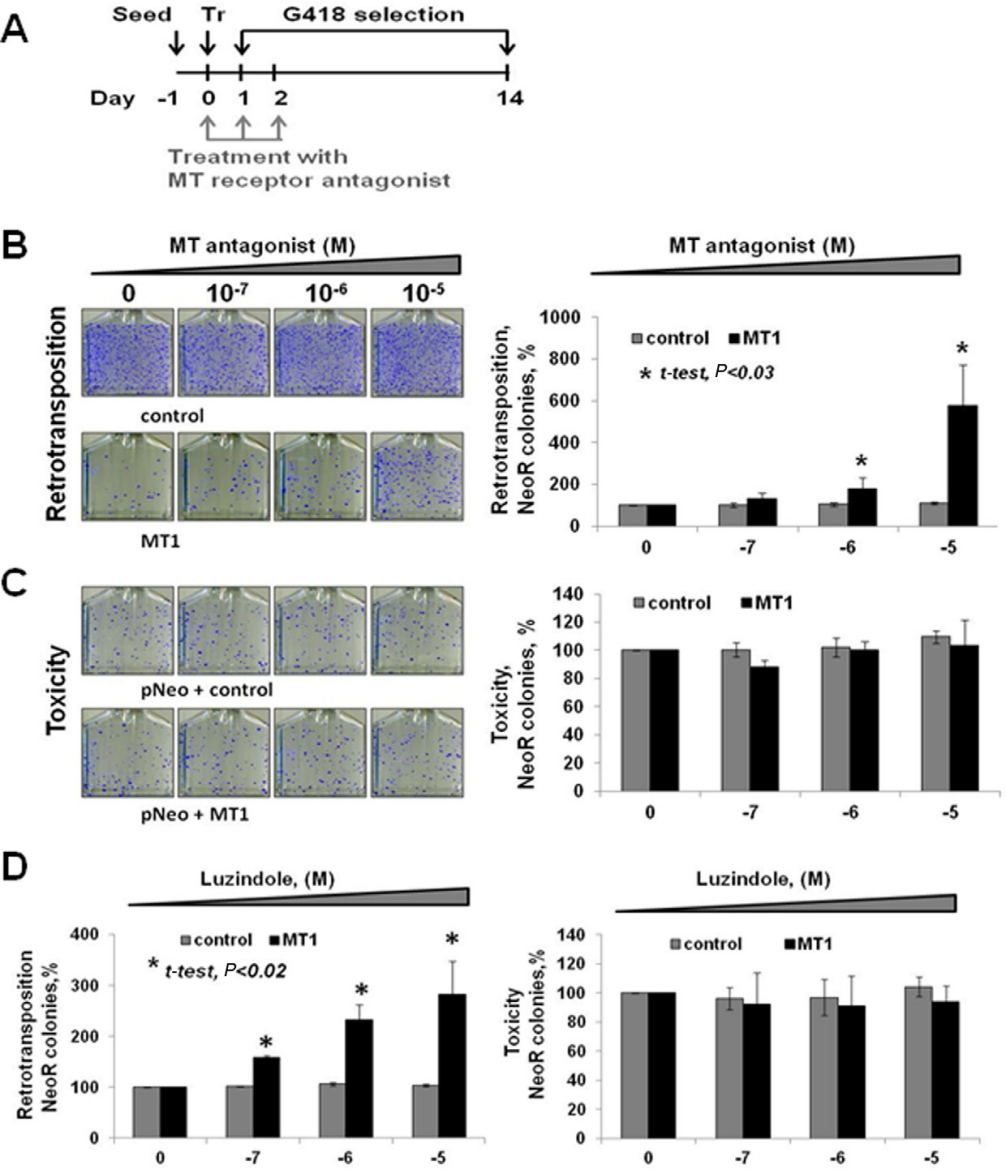
MT1 effect on L1 retrotransposition is specific to MT1 function. (**A**) Experimental approach for treatment with melatonin receptor antagonists. Treatment with increasing doses of melatonin receptor antagonist was initiated on the day of transfection (Day 0) and was performed two additional times (Day 1 and 2). G418 selection was initiated on Day 1 and continued for 14 days. (**B**) Melatonin receptor antagonist S20928 relieves MT1 suppression of L1 retrotransposition in HeLa cells in a dose-dependent manner. L1 retrotransposition in HeLa cells in the presence of the control plasmid is not affected by melatonin receptor antagonist treatment (left panel top row and right panel gray bars), but it is increased when the treatment is combined with MT1 expression (left panel bottom row and right panel black bars). Colony numbers obtained for retrotransposition and toxicity without any treatment with melatonin receptor antagonist are set as 100%. (**C**) MT1 expression and melatonin receptor antagonist S20928 treatment do not affect cell viability and colony formation in HeLa cells. (**D**) Melatonin receptor antagonist luzindole relieves MT1 suppression of L1 retrotransposition in HeLa cells without any adverse effect on cell viability and colony formation. Error bars are standard deviation; asterisks indicate statistically significant differences by *t*-test.

To confirm the specificity of MT1 effect on L1 and Alu retrotransposition, we used melatonin receptor antagonists (S20928 or luzindole). Treatment with increasing doses of MT1 antagonists was performed once a day for the first 3 days of the retrotransposition assay starting at the time of transfection cocktail removal (Figure 2A). This experimental scheme was used because prolonged treatments with melatonin receptor antagonists are toxic and the bulk of L1 retrotransposition occurs during the first 2–3 days after transfection (50). Treatments with increasing doses of melatonin receptor antagonist S20928 have no effect on L1 retrotransposition when the Neo-tagged L1 expression plasmid was cotransfected with an empty control vector (Figure 2B left panel top row and right panel gray bars). The same treatments significantly increased L1 retrotransposition in HeLa cells in a dose-dependent manner when the same L1 expression plasmid was cotransfected with the MT1 expression plasmid (Figure 2B left panel bottom row and right panel black bars). No toxicity was observed when the cells were treated with S20928 antagonist alone or in combination with L1 and MT1 (Figure 2C). Treatment of L1 and MT1 transfected HeLa cells with another melatonin receptor antagonist luzindole resulted in a similar dose-dependent increase in L1 retrotransposition (Figure 2D, left panel black bars). Again, no adverse effect of melatonin antagonist luzindole treatment was detected by the toxicity assay (Figure 2D, right panel black bars).

The same approach was used to test the effect of melatonin receptor antagonist treatment on L1-driven Alu retrotransposition (Supplementary Figure S3A). Similar to L1, treatments with luzindole increased L1-driven Alu retrotransposition in a dose-dependent manner (Supplementary Figure S3A, black bars) without any adverse effect on cell viability and colony formation (Supplementary Figure S3B).

### MT1 overexpression reduces L1 mRNA and ORF1 protein in cultured cells

**Figure 3.**
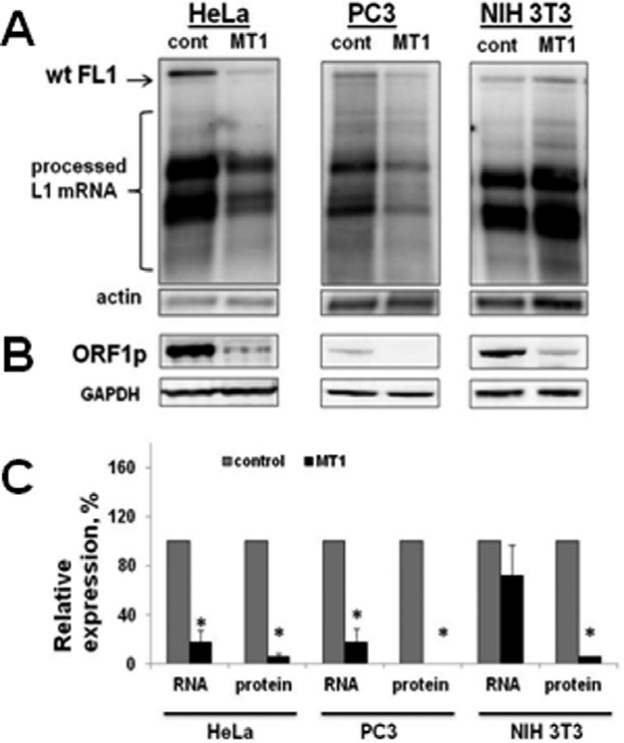
The effect of MT1 overexpression on L1 RNA and ORF1p. (**A**) Northern blot analysis of L1 mRNA in HeLa, PC3 and NIH 3T3 cells transiently transfected with the wild-type human L1 expression plasmid and the control or MT1 expression plasmids. Northern blot analysis was carried out with a strand-specific RNA probe complementary to the 100 bp region in the beginning of the L1 5′UTR (28,30). wt FL1 indicates the full-length L1 mRNA; processed L1 mRNA indicates prematurely polyadenylated and spliced L1-related mRNAs (28,30). Actin is actin mRNA. (**B**) Western blot analysis of L1 ORF1p in HeLa, PC3 and NIH 3T3 cells transiently transfected with the wild-type human L1 expression plasmid and the control or MT1 expression plasmids. GAPDH (Glyceraldehyde 3-phosphate dehydrogenase)detection was used as loading control. (**C**) Quantitation of L1 mRNA (full-length) and ORF1p in different cell lines. Asterisks indicate statistical significance between L1 mRNA and ORF1p detected in the same cell line in the presence or absence of melatonin receptor expression (*t*-test, *P* < 0.05). Actin and GAPDH were used for normalization of the RNA and protein signals, respectively. Error bars are standard deviation.

To understand the mechanism of MT1 downregulation of L1 retrotransposition, we tested the effect of MT1 overexpression on the steady-state levels of L1 mRNA and ORF1p. L1 mRNA was measured in HeLa, PC3 and NIH 3T3 cells cotransfected with the untagged human L1 expression plasmid and either empty or MT1 expression plasmids (Figure 3A). Northern blot analysis with a strand-specific RNA probe complementary to the first 100 bp of the L1 5′UTR (28) demonstrated a significant decrease in L1 mRNA levels in HeLa and PC3 cells overexpressing human MT1 receptor compared to the control cells transfected with an empty vector (Figure 3A and C). This effect was less pronounced in NIH 3T3 cells in which full-length L1 mRNA produced by the transiently transfected L1 expression plasmid appears to be more stable (Supplementary Figure S4). Parallel analysis of ORF1p expression in the same cell types using ORF1p-specific antibody (51) demonstrated significant reduction in the ORF1p levels in all three cell lines (Figure 3B and C). The same reduction of steady-state L1 ORF1p in the presence of MT1 receptor was observed in immortalized human fibroblasts (Supplementary Figure S5).

**Figure 4.**
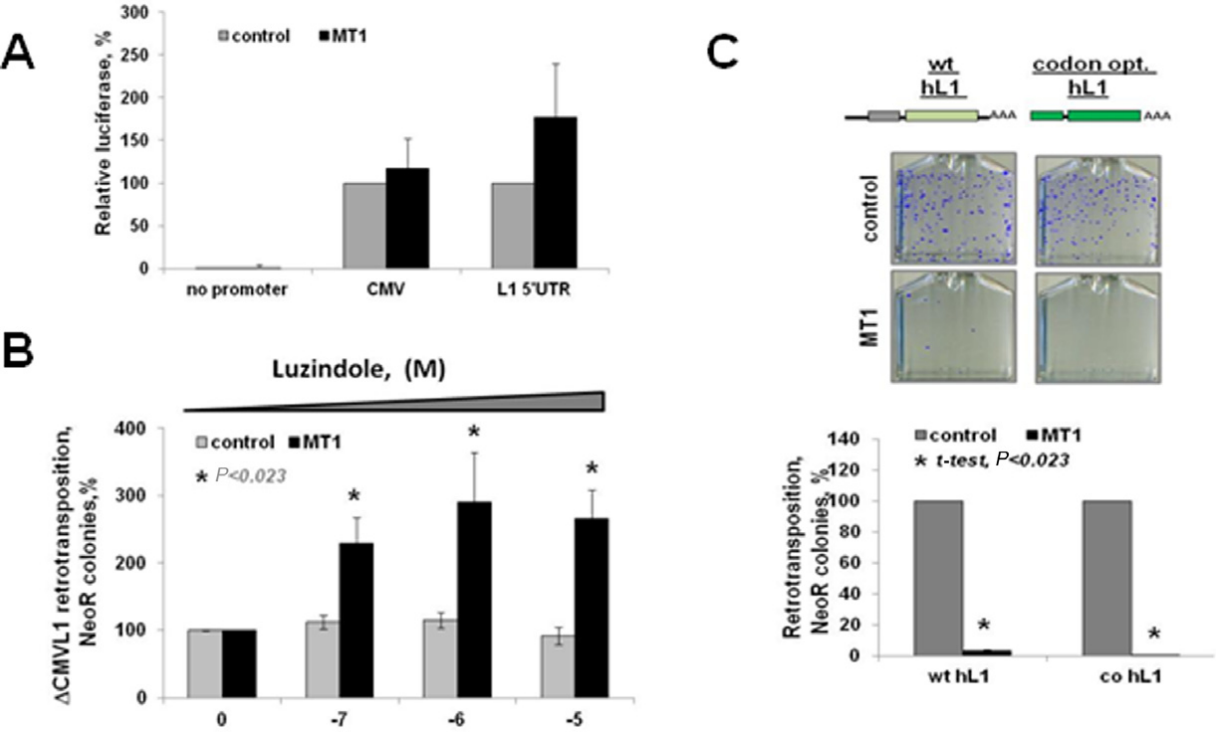
MT1-induced suppression of retrotransposition is independent of the L1 promoter. (**A**) Fluc activity was assessed in HeLa cells transiently transfected with Fluc expression vector driven by CMV or L1 promoters and cotransfected with either an empty (control, gray bars) or MT1 expression plasmids (MT1, black bars). A vector expressing Renilla luciferase was used as transfection control. Fluc vector without any promoter was used as negative control (no promoter). Luciferase activity is shown as percent of the activity detected in cotransfection with empty plasmid for each construct. (**B**) Luzindole treatment eliminates the downregulatory effect of MT1 overexpression on L1 retrotransposition when L1 is driven by its own promoter (ΔCMVL1) (also Supplementary Figure S7A). (**C**) Alu retrotransposition driven by the wild-type human L1 expression plasmid (wt hL1) or by the codon-optimized human L1 (codon opt and co hL1) expression plasmid with and without cotransfection with the MT1 expression plasmid. Error bars are standard deviation; asterisks indicate statistically significant differences by *t*-test.

Reduced levels of L1 mRNA could be due to an effect of MT1 on the promoter driving L1 expression. The L1 and CMV promoters drive the L1Neo and untagged L1 constructs used in the above-described experiments. Expression of a Firefly luciferase (Fluc) driven by the L1 (52) or CMV (Promega) promoters demonstrated that MT1 overexpression does not have any significant effect on *Fluc* activity driven by either promoter (Figure 4A). Furthermore, MT1 overexpression had the same effect on L1 retrotransposition whether L1 expression was driven by the CMV promoter alone or by the native L1 promoter (Supplementary Figure S6, CMV/ΔL1Blast and ΔCMV/L1Neo). A dose-dependent increase in L1 retrotransposition in the presence of MT1 expression and luzindole treatment was observed when L1 expression was driven by its own promoter (ΔCMVL1Neo) (Figure 4B, black bars). The same result was observed when untagged L1 driven by its own promoter (ΔCMV L1) was used to support Alu retrotransposition (Supplementary Figure S7, black bars).

Another plausible mechanism by which MT1 overexpression may reduce L1 mRNA levels is through the alteration of L1 mRNA stability in a sequence-dependent manner. To identify potential *cis*-acting sequences within the L1 mRNA that might be responding to MT1, we used a human L1 expression vector lacking L1 5′ and 3′ UTRs and containing codon-optimized sequences (50,67) to drive Alu retrotransposition (Figure 4C). Regardless of the changes introduced to L1, Alu mobilization was downregulated when cotransfected with MT1, supporting that the effect of MT1 on retrotransposition is independent of the primary L1 sequence (Figure 4C, MT1, black bars).

### Inactivating mutations of putative phosphorylation and ubiqutination sites within ORF1 protein abolish MT1 effect

**Figure 5.**
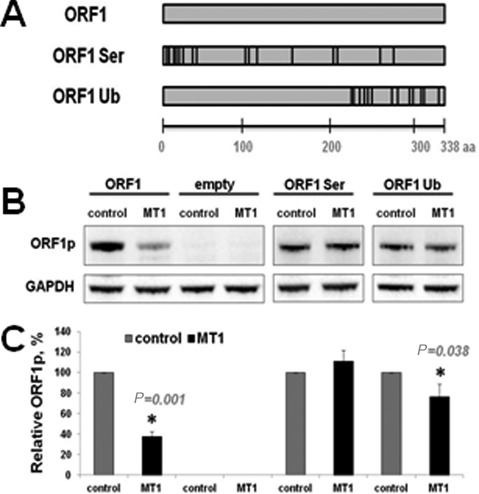
Inactivating mutations of putative phosphorylation and ubiquitination sites predicted within ORF1p sequence abolish MT1 effect on ORF1p. (**A**) Schematic of putative phosphorylation (ORF1 Ser) and ubiquitination (ORF1 Ub) sites predicted within the ORF1p sequence mutated to alanine. (**B**) Western blot analysis of ORF1p in the presence or absence of melatonin receptor 1 expression (MT1 and control, respectively). Transfection with an empty vector was used to assess background levels of ORF1p. GAPDH is used as loading control. (**C**) Quantitation of western blot results (*N* = 3). ORF1, ORF1 Ser and ORF1 Ub expression in the presence of MT1 (black bars) is normalized to their respective expression in the presence of control vector (gray bars). Error bars are standard deviation; asterisks indicate statistically significant differences between control and MT1 lanes by *t*-test.

It is established that multiple signaling pathways can influence cellular protein levels by phosphorylation followed by degradation (68). NetPhos 2 and CKSAAP prediction programs were used to identify putative phosphorylation and ubiquitination sites in the ORF1p sequence (54,55). The programs identified 20 putative phosphorylation and 32 putative ubiqutination sites in the L1 ORF1p. Sixteen and 14 of these phosphorylation (serine only) and ubiqutination sites, respectively, with highest predicted probability values were mutated to alanines to generate ORF1 Ser and ORF1 Ub mutants (Figure 5A). Western blot analysis of total cell lysates collected from HeLa cells expressing ORF1, ORF1 Ser and ORF1 Ub proteins in the presence or absence of MT1 demonstrated that serine to alanine mutations completely abolished the suppressive effect of MT1 expression (Figure 5B and C, ORF1 and ORF1 Ser). ORF1 Ub protein retained minimal sensitivity to MT1 overexpression (Figure 5B and C, ORF1 Ub). Combined these data predict that L1 expression is expected to be suppressed by nocturnal production of melatonin and its receptor *in vivo*.

### Melatonin through its receptor regulates endogenous L1 mRNA in a prostate cancer model *in vivo*

**Figure 6.**
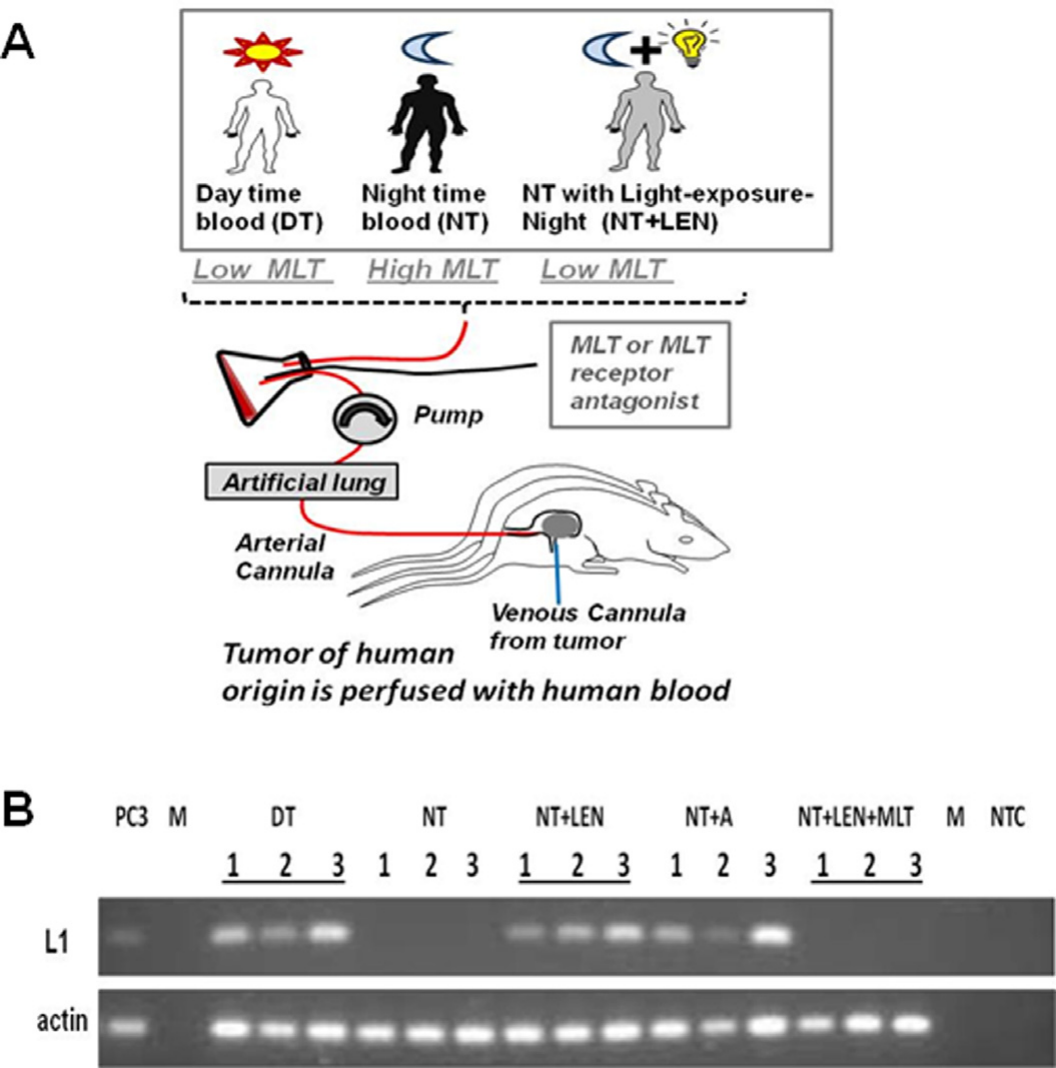
Endogenous L1 expression is regulated by melatonin in prostate cancer model *in vivo*. (**A**) Experimental design. Human prostate tissue-isolated xenografts were established in nude male rats as described (56,69). Animals were housed under normal light exposure (12L:12D). Whole blood was collected from the same healthy male donors during the day (DT), night (NT) or at night after exposure to light (NT+LEN, LEN is light exposure at night) and used for *in situ* perfusion of human prostate xenografts as described (58). MLT is melatonin. (**B**) Analysis of endogenously expressed L1 mRNA. Tumor samples are labeled as described in (A). NT+A is nighttime blood supplemented with melatonin receptor antagonist (S20928) during *in situ* perfusion. NT+LEN+MLT is melatonin-low blood collected at night after exposure to light and supplemented with exogenous melatonin during *in situ* perfusion. RT-PCR is performed with primers amplifying a region within 1–100 bp of the human L1 5′UTR, L1, (see Supplementary Figure S6). PC3 are prostate cancer cells used to generate tissue-isolated xenografts, M is a marker lane and NTC is a no template control. 1, 2, 3 are the same three donors for each time point. Actin is used as loading control.

To test circadian regulation of L1 *in vivo*, we used a tissue-isolated xenograft model of human prostate cancer originating from PC3 prostate cancer cells used in above-described experiments. The tumors were established in nude male rats housed under normal light conditions (12L:12D) (69) and perfused *in situ* as described (57) with human blood collected from adult male donors either during day time (DT, low melatonin levels), night time (NT, high melatonin levels) or night time after exposure to bright light (LEN, low melatonin levels) (58) (Figure 6A). RT-PCR primers specific to the human L1 element were used to analyze endogenous L1 expression in tumors generated in this prostate cancer model (Supplementary Figure S8A and B). RT-PCR analysis was performed with RT-control for each time point and each donor. Supplementary Figure S8C shows RT-PCR results with appropriate controls for one donor. Figure 6B demonstrates a summary of the results collected from different donors. RT-PCR analysis demonstrated that endogenous L1 mRNA is significantly downregulated in tumors perfused with melatonin-rich blood (Figure 6B, NT versus DT, and Supplementary Figure S8C), supporting the above-formulated hypothesis that L1 expression is suppressed during the dark phase of the photoperiod. In contrast to NT blood, perfusion of tumors with blood collected from the same donors after 1-h exposure to bright light at night (2800 lx, NT+LEN, which suppresses endogenous melatonin production) did not result in the reduction of L1 mRNA (Figure 6B, NT+LEN).

To confirm that the observed effect is due to the changes in melatonin levels among the daytime, nighttime and nocturnal light exposure (LEN) blood samples, melatonin receptor antagonist S20928 was added during *in situ* perfusion to the blood collected at night. The addition of this antagonist abolished the inhibitory effect of the melatonin-rich NT blood on L1 mRNA (Figure 6B, NT+A and Supplementary Figure S7C). Likewise, supplementation of blood that has very low endogenous melatonin levels with exogenous melatonin during *in situ* perfusion suppresses L1 mRNA in a manner similar to the nighttime, melatonin-rich blood (Figure 6B, NT, NT+LEN, and LEN+MLT and Supplementary Figure S7C). No endogenous ORF1p signal was detected in any of the samples analyzed for RNA expression using our ORF1p-specific antibodies.

## DISCUSSION

In 2007, the World Health Organization recognized light exposure at night as a probable carcinogen (47). This recognition is based on several epidemiological studies (70,71) and research using animal models (32,72) that demonstrate that shift workers and animals exposed to light at night have increased risk of cancer. While the carcinogenic nature of this light exposure is appreciated, the physiology and molecular mechanisms underlying this phenomenon are not fully understood.

Despite the fact that human cancers differ significantly in their time of onset, aggressiveness and response to treatment, it is well established that they share at least one common feature (and their ultimate root cause), which is genomic instability. Genomic instability in the form of point mutations, insertions and deletions, as well as large chromosomal rearrangements is responsible for cancer initiation, progression and heterogeneity [reviewed in (73)]. Genomic instability can be caused by intrinsic factors or be induced by environmental exposures. One of the intrinsic DNA-damaging agents that can cause different types of genomic instability often found in human cancers is L1 retrotransposon [reviewed in (16)], expression of which is usually upregulated in many human cancers compared to their matching normal tissues (3,74,75). Recent second-generation sequencing analyses of *de novo* L1 retrotransposition in several human cancers demonstrated that some of these inserts occurred early in cancer development (5,22,24,76). Most interestingly, a proportion of *de novo* L1 integrations disrupted normal expression of cancer-relevant genes, supporting L1's role in generation of genomic alterations relevant to cancer origin, progression and heterogeneity (22). These studies also found that the number of *de novo* L1 inserts varies significantly even among tumors (5,22) of the same type, which could arise from the difference in the number of functional polymorphic L1 loci in the human population (77,78) or genetic variation in cellular DNA repair (29,79). The recognition of light exposure at night as a cancer risk factor raises a question of how this environmental stimulus can promote different types of cancer. One possibility may be that light exposure at night promotes genomic instability. If so, upregulation of L1 expression and activity by environmental light exposure at night could be one of the contributing factors driving genomic instability relevant to cancer risk and/or progression in shift workers.

We demonstrate that expression of endogenous L1 elements in a tissue-isolated model of prostate cancer is suppressed by melatonin circulating in human blood (Figure 6 and Supplementary Figure S8). This regulation is disrupted by the host's exposure to light at night, which suppresses nocturnal melatonin production (42). Indeed, melatonin-depleted blood collected after exposure to light at night does not reduce endogenous L1 mRNA as does melatonin-rich blood collected during the dark phase of the circadian cycle. Circulating melatonin suppresses endogenous L1 mRNA in a receptor-mediated manner (Figure 6), which is demonstrated by the abolishment of the effect of melatonin on endogenous L1 mRNA levels in a prostate cancer model by the addition of MT1 and MT2 antagonist during *in situ* tumor perfusion (Figure 6). Consistent with this result, the addition of exogenous melatonin during *in situ* perfusion restores suppression of endogenous L1 mRNA, suggesting that the effect of light exposure at night on L1 mRNA in tumors can be mitigated by melatonin supplementation.

Consistent with our *in vivo* observation, overexpression of MT1 receptor decreases steady-state levels of L1 mRNA and L1 ORF1p in cultured cells (Figure 3 and Supplementary Figure S5) and suppresses L1 retrotransposition (Figures 1 and 2). Administration of melatonin receptor antagonists during the retrotransposition assay increases L1 and Alu mobilization in a dose-dependent manner (Figure 2 and Supplementary Figures S3 and S7). This effect is independent of the L1 promoter (Figure 4A and Supplementary Figure S6) and L1 mRNA sequence (Figure 4C) as Alu retrotransposition by the L1 containing codon-optimized ORF1 and ORF2 sequences and lacking its 5′ and 3′ UTRs is still suppressed by melatonin receptor overexpression (Figure 4C). Furthermore, MT1 suppresses ORF1p even when it is expressed by a codon-optimized plasmid containing only ORF1 sequence (Figure 5). In contrast, L1 ORF1ps with mutations of putative phosphorylation or ubiquitination sites are no longer affected by the MT1 overexpression, even though their expression is driven by the same promoter as the one driving the expression of the wild-type protein (Figure 5, ORF1 Ser and ORF1 Ub).

Many signaling pathways, including melatonin signaling, activate a cascade of protein kinases that decrease amounts of cellular proteins by targeting them for degradation or change their ability to associate with their interacting partners (68,80). Indeed, Akt/PKB signaling is reported to decrease ischemia-induced activation of L1 expression in rat heart (81) and many cellular proteins have been recently reported to be associated with L1 ORF1p (82). The ORF1p forms homotrimers, which associate with the L1 mRNA (8,10,11,83) to form RNPs. This association potentially plays a role in protecting the L1 mRNA from degradation or enabling its dissociation from the polyribosomes. Little is known about the regulation of this ORF1p self-association and RNP formation in mammalian cells (84,85). Melatonin signaling may trigger postranslational modifications altering ORF1p stability or its ability to interact with itself or cellular proteins. We demonstrate that MT1 suppresses ORF1p even when the protein is expressed outside of the context of the full-length L1 (Figure 5). ORF1p contains numerous putative phosphorylation and ubiquitination sites identified by respective prediction programs (Figure 5). If there is any cooperation between these sites in the MT1-induced downregulation of L1 ORF1p, then independent elimination of either putative phosphorylation or ubiquitination sites from ORF1p sequence should dampen its response to MT1. We demonstrate that inactivating point mutations of putative phosphorylation or ubiquitination sites within ORF1p abolish negative effect of MT1 on ORF1p expression (Figure 5). These ORF1p mutants suggest a mechanism of MT1-induced phosphorylation of L1 ORF1p, which likely targets it for degradation through proteasomal or proteolytic degradation. Furthermore, the decrease in L1 ORF1p may reduce L1 mRNA stability due to the disruption of RNP formation. Alternatively, it is also possible that point mutations introduced into the ORF1p sequence disrupt protein:protein interactions important for ORF1p stability. Identifying which specific mutations within the ORF1p sequence are responsible for the MT1 effect of L1 ORF1p and the degree of redundancy in the ORF1p response to MT1 will refine our understanding of this mechanism and the cellular players responsible for its action. Likewise, further studies exploring the hypothesis of light exposure at night increasing L1 retrotransposition *in vivo* will be needed to determine whether the genomes of shift workers or other individuals who have experienced extensive LEN accumulate increased amounts of L1-induced damage.

**Figure 7.**
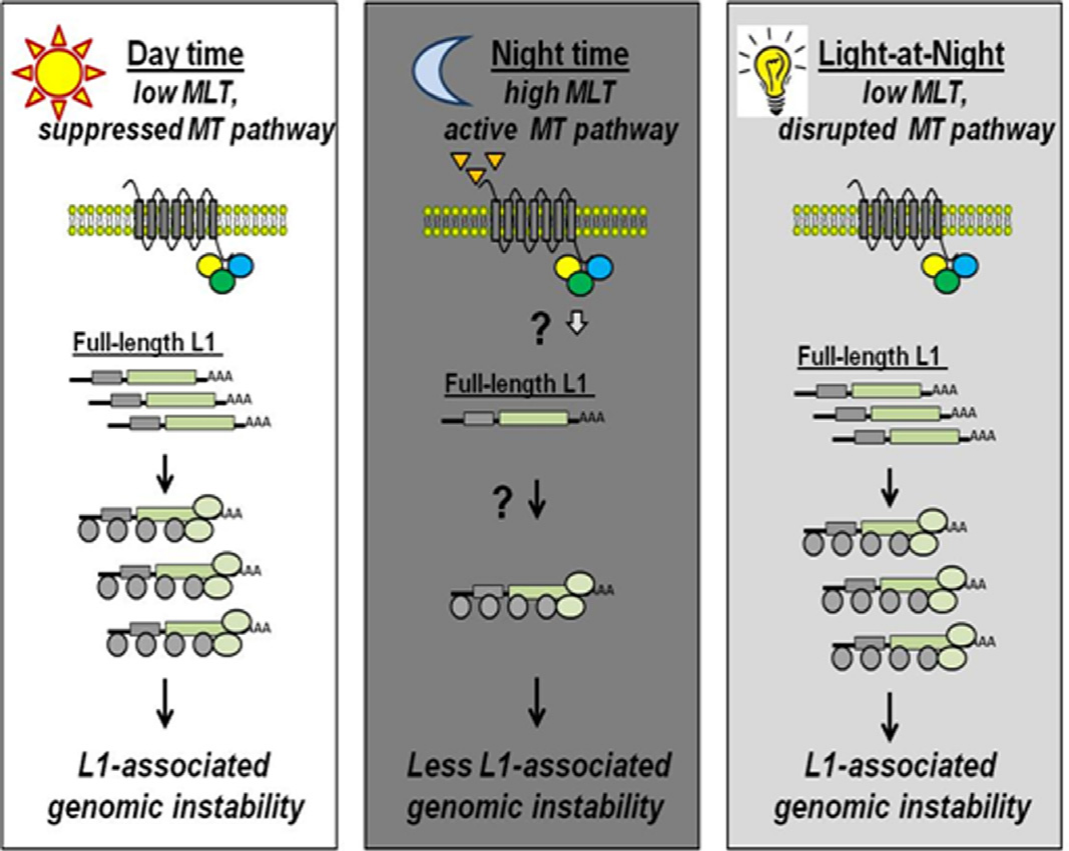
A model for regulation of L1 expression and damage by environmental light exposure. During the day when melatonin production and secretion as well as melatonin receptor expression is suppressed by light, L1 mRNA and proteins are expected to accumulate leading to L1-induced damage (left panel). During the night, ongoing melatonin production activates melatonin receptor, resulting in the suppression of L1 mRNA and ORF1p, consequently leading to a decrease in L1-associated genomic instability (middle panel). Exposure to light at night, which encompasses subpopulations of shift workers, people with sleeping disorders, caretakers and the elderly, disrupts normal melatonin production allowing accumulation of L1 mRNA and proteins that leads to L1-induced damage during the time when the host genome normally would be protected from L1 DNA-damaging activity (right panel).

Combined, our *in vivo* data and tissue culture-based results support the following model of light-dependent regulation of L1 in cancer. Under normal light exposure, nocturnal melatonin production activates melatonin receptors, leading to suppression of L1 mRNA and ORF1p (Figure 7). Nighttime light exposure suppresses nocturnal melatonin production, which abolishes activation of melatonin receptor(s) signaling needed to suppress L1 mRNA. Based on these data, we propose that suppression of nocturnal melatonin production by light results in accumulation of L1 mRNA and ORF1p in tumor cells during the night when the genome is normally protected from L1 activity (Figure 7). This continuous L1 expression in tumors likely results in an increase in L1 retrotransposition *in vivo,* leading to accumulation of *de novo* integration events in the tumors of shift workers or individuals with disrupted melatonin production. While specific molecular events controlling regulation of L1 by melatonin have yet to be identified, this finding provides a tantalizing first glimpse into the complex regulation of L1 activity *in vivo*. The connection between the circadian system and L1 suggests that L1-induced damage is likely increased in tumors originating in shift workers and the elderly. Both subpopulations have reduced nocturnal melatonin levels and increased incidence of cancer (43,44,86,87) that could partly be due to the upregulation of somatic L1 activity reported to introduce cancer-driving mutations (22,23).

Our findings also raise important questions about the control of L1 expression in normal tissues. We have previously detected various amounts of endogenous L1 mRNA in a broad spectrum of normal human tissues (3). This variation can occur because of the tissue-specific differences in premature polyadenylation and splicing of L1 transcripts (28,30). The observation that L1 mRNA is regulated by the circadian system in tumors also supports circadian regulation of L1 expression in normal tissues. Thus, the reported differences in the amount of the full-length L1 mRNA could also result from the collection of these tissues at different times during the circadian cycle. While no experimental evidence supporting L1 regulation by the host circadian system in normal tissues exists, our data predict that L1 expression in normal tissues would demonstrate a 24-h periodicity. In contrast to most tumors that are often deficient in the function of core circadian genes (88,89), but maintain melatonin receptor function (38), normal tissues express functional core circadian proteins and are responsive to the nocturnal melatonin production. As a result, the pattern of L1 expression over a 24-h period in normal tissues may differ from that found in tumors. L1-induced damage in normal tissues is also expected to be affected by melatonin in a tissue-specific manner due to the variation in MT1 expression (35). Furthermore, L1 damage can be regulated in a circadian manner because of the oscillation in expression or activity of cellular proteins and pathways known to influence L1 expression or steps of the L1 integration process, such as the nucleotide excision repair (NER) pathway (33). NER is regulated by the circadian system and has been shown to suppress L1 retrotransposition in cultured cells (29,33).

The implications of circadian regulation of L1 expression and activity expand beyond its impact on the genomes of shift workers. Our findings suggest a possibility of an increase in L1-induced genomic instability with age. An age-dependent decline in melatonin production and melatonin receptor expression may result in upregulation of L1 expression, leading to an increase in L1 activity and the mutagenic burden associated with its expression. Consistent with this idea is a recent study on somatic L1 retrotransposition in colorectal cancers, which found a strong correlation between L1 retrotransposition and age (5). Furthermore, age-associated circadian decline may enhance L1-induced genomic instability not only through a direct increase in L1 expression, but also via the loss of DNA repair response necessary to shield the genome from L1 damage (90,91).

Overall, our finding of receptor-mediated melatonin-induced suppression of L1 expression and activity adds an important component to the numerous existing cellular pathways known to downregulate L1 expression and activity. It establishes a link among the host circadian system, environmental light exposure and genomic instability. Furthermore, this discovery has important evolutionary implications. Melatonin is one of the most evolutionarily conserved molecular entities, estimated to originate some 2.5 billion years ago (92). Transposable elements, particularly retrotransposons, can be found in animals, plants, protozoans and fungi (93), suggesting that melatonin and the circadian system may be a common pathway of suppression or regulation of these elements in different kingdoms.

In conclusion, L1 elements and their damage *in vivo* should be considered as a dynamic, probably age-dependent, entity with effects consequential to the whole human body, but likely unique to individual tissues, genetic backgrounds and environmental light exposures.

## SUPPLEMENTARY DATA

Supplementary Data are available at NAR Online.

SUPPORTING INFORMATION
