# Supplementary material for: Regulation of L1 expression and retrotransposition by melatonin and its receptor: implications for cancer risk associated with light exposure at night
Source: Nucleic Acids Res. 2014 Jun 9;42(12):7694–707. doi: 10.1093/nar/gku503 (PMC4081101; doi:10.1093/nar/gku503)
Supplement: SUPPORTING INFORMATION [file supp_42_12_7694__index.html]

Regulation of L1 expression and retrotransposition by melatonin and its receptor: implications for cancer risk associated with light exposure at night — SUPPORTING INFORMATION 

# Regulation of L1 expression and retrotransposition by melatonin and its receptor: implications for cancer risk associated with light exposure at night

## SUPPORTING INFORMATION

**Files in this Data Supplement:**

- Supplemental Figures
