# Supplementary material for: Regulation of L1 expression and retrotransposition by melatonin and its receptor: implications for cancer risk associated with light exposure at night
Source: Nucleic Acids Res. 2014 Jun 9;42(12):7694–707. doi: 10.1093/nar/gku503 (PMC4081101; doi:10.1093/nar/gku503)
Supplement: SUPPORTING INFORMATION [file supp_gku503_nar-02612-a-2013-File009.pptx]

## Slide 1
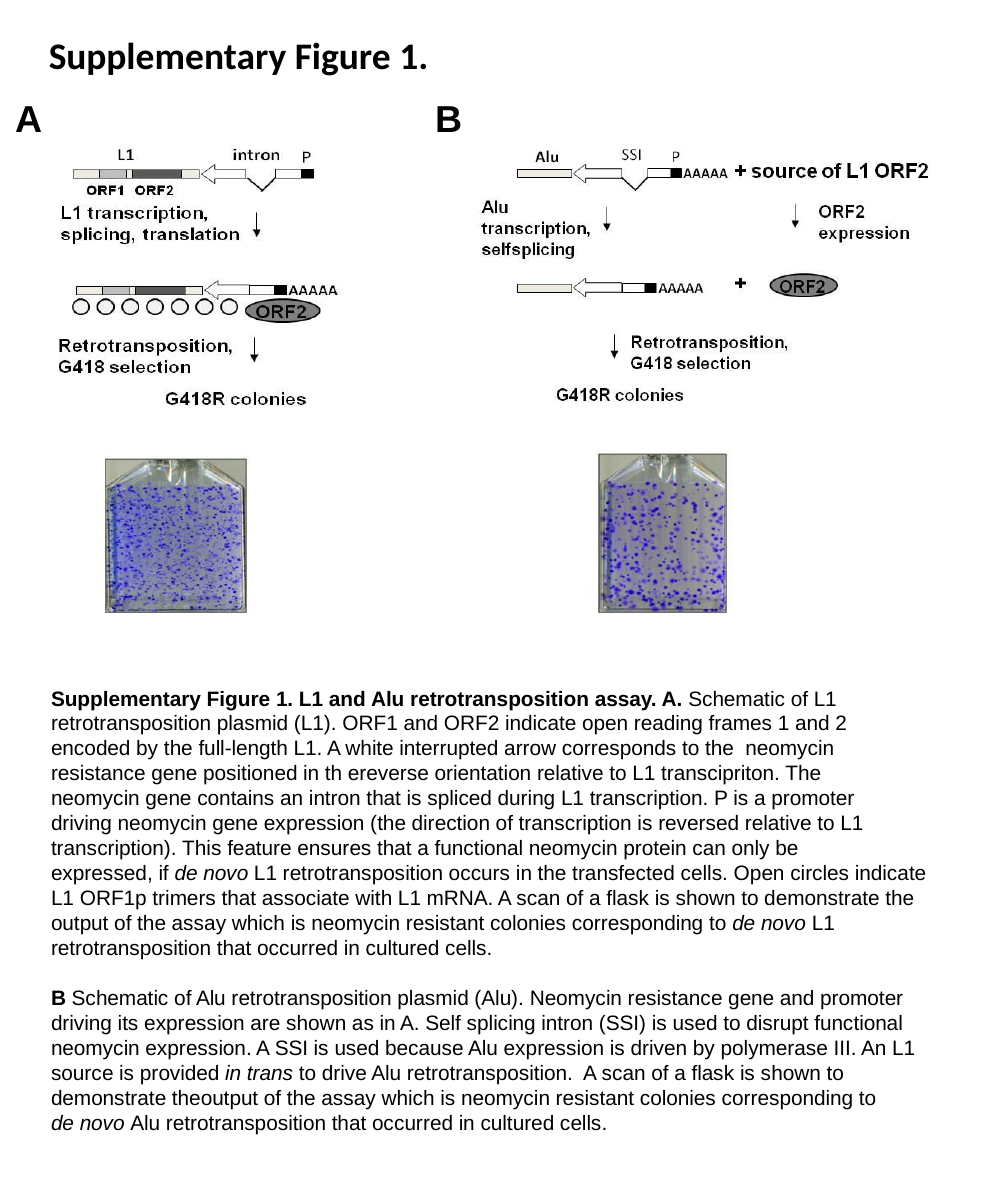

Supplementary Figure 1.
A
B
Supplementary Figure 1. L1 and Alu retrotransposition assay. A. Schematic of L1
retrotransposition plasmid (L1). ORF1 and ORF2 indicate open reading frames 1 and 2
encoded by the full-length L1. A white interrupted arrow corresponds to the neomycin
resistance gene positioned in th ereverse orientation relative to L1 transcipriton. The
neomycin gene contains an intron that is spliced during L1 transcription. P is a promoter
driving neomycin gene expression (the direction of transcription is reversed relative to L1
transcription). This feature ensures that a functional neomycin protein can only be
expressed, if de novo L1 retrotransposition occurs in the transfected cells. Open circles indicate
L1 ORF1p trimers that associate with L1 mRNA. A scan of a flask is shown to demonstrate the
output of the assay which is neomycin resistant colonies corresponding to de novo L1
retrotransposition that occurred in cultured cells.
B Schematic of Alu retrotransposition plasmid (Alu). Neomycin resistance gene and promoter
driving its expression are shown as in A. Self splicing intron (SSI) is used to disrupt functional
neomycin expression. A SSI is used because Alu expression is driven by polymerase III. An L1
source is provided in trans to drive Alu retrotransposition. A scan of a flask is shown to
demonstrate theoutput of the assay which is neomycin resistant colonies corresponding to
de novo Alu retrotransposition that occurred in cultured cells.

## Slide 2
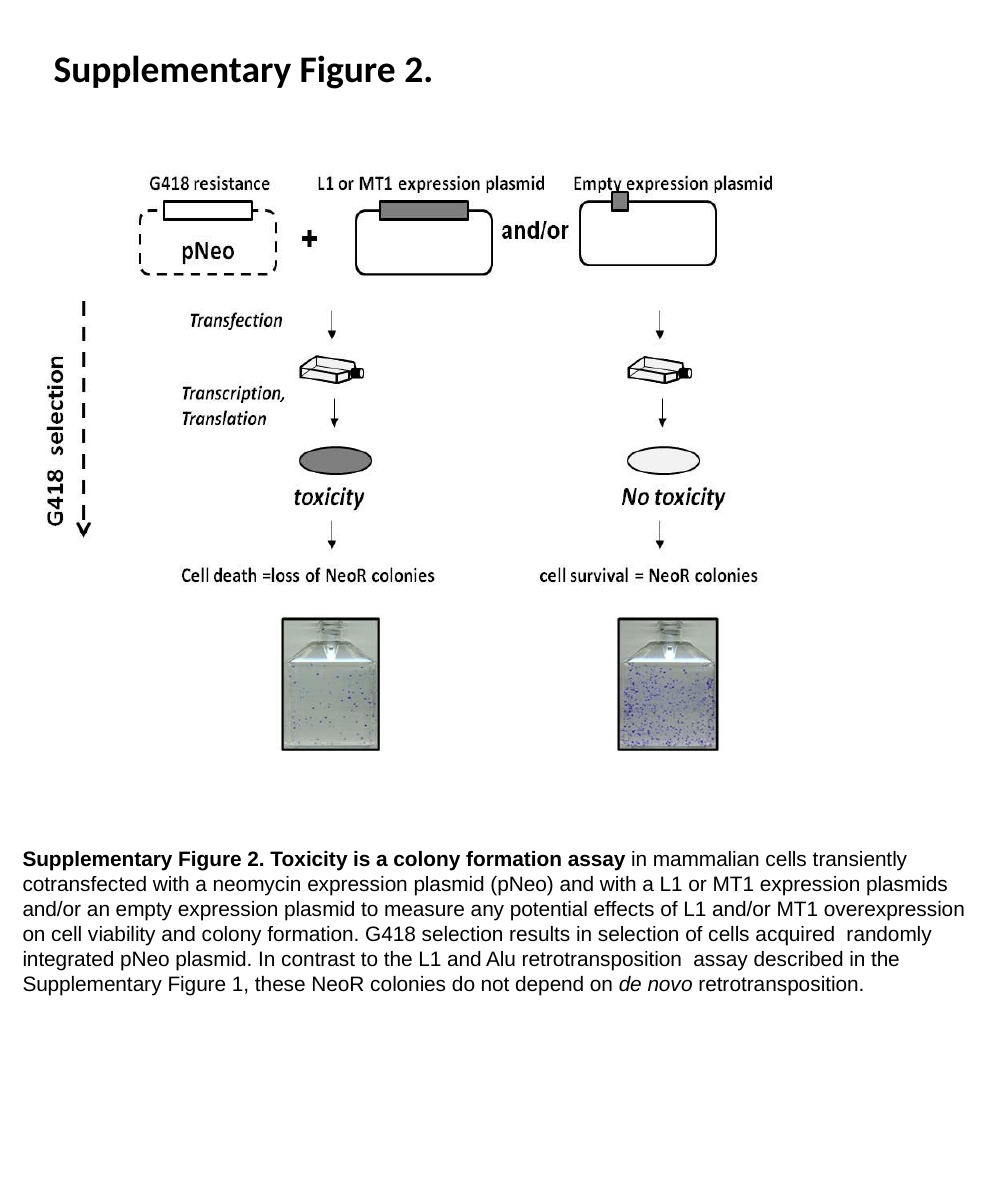

Supplementary Figure 2.
Supplementary Figure 2. Toxicity is a colony formation assay in mammalian cells transiently
cotransfected with a neomycin expression plasmid (pNeo) and with a L1 or MT1 expression plasmids
and/or an empty expression plasmid to measure any potential effects of L1 and/or MT1 overexpression
on cell viability and colony formation. G418 selection results in selection of cells acquired randomly
integrated pNeo plasmid. In contrast to the L1 and Alu retrotransposition assay described in the
Supplementary Figure 1, these NeoR colonies do not depend on de novo retrotransposition.

## Slide 3
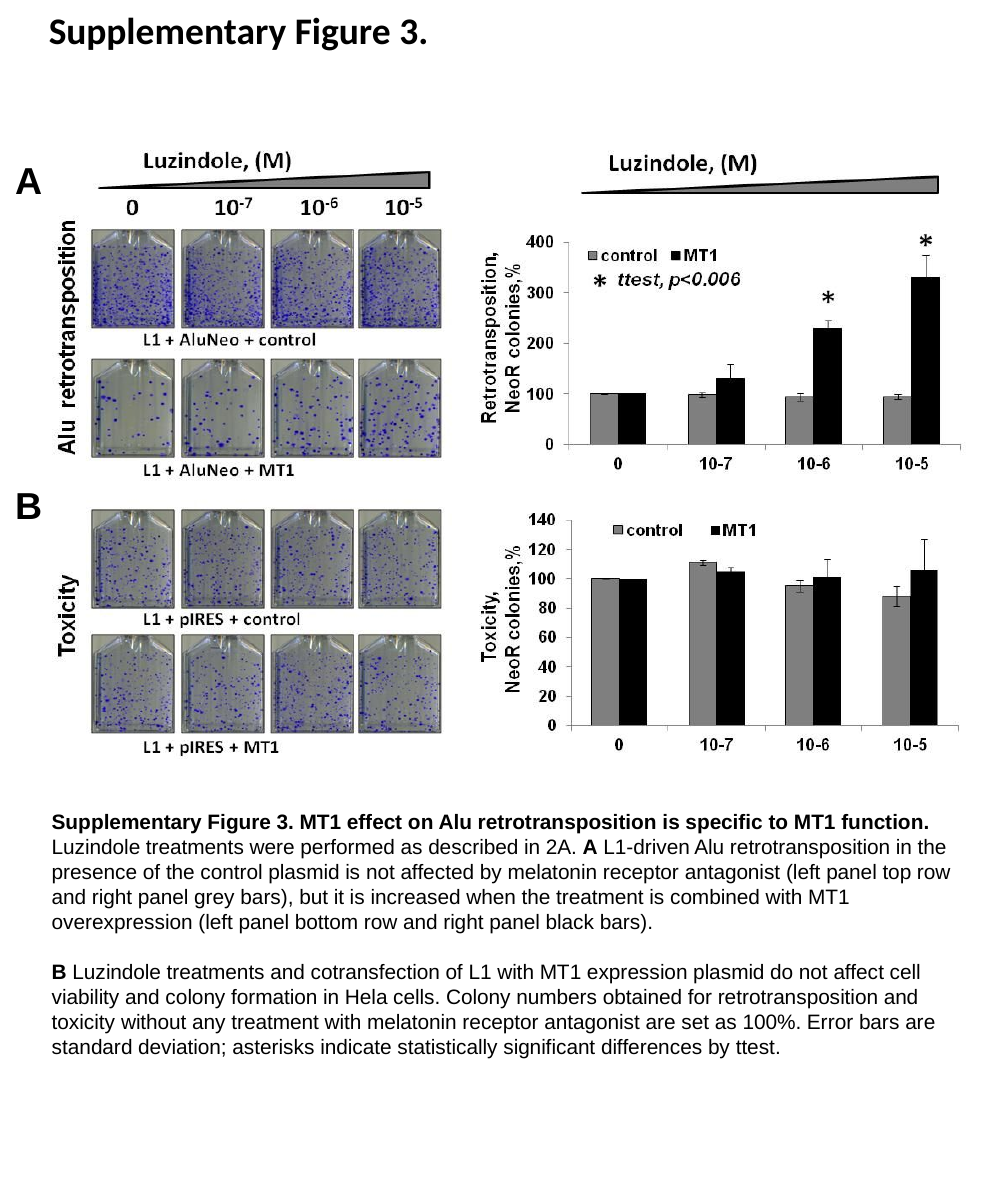

Supplementary Figure 3.
A
B
Supplementary Figure 3. MT1 effect on Alu retrotransposition is specific to MT1 function.
Luzindole treatments were performed as described in 2A. A L1-driven Alu retrotransposition in the
presence of the control plasmid is not affected by melatonin receptor antagonist (left panel top row
and right panel grey bars), but it is increased when the treatment is combined with MT1
overexpression (left panel bottom row and right panel black bars).
B Luzindole treatments and cotransfection of L1 with MT1 expression plasmid do not affect cell
viability and colony formation in Hela cells. Colony numbers obtained for retrotransposition and
toxicity without any treatment with melatonin receptor antagonist are set as 100%. Error bars are
standard deviation; asterisks indicate statistically significant differences by ttest.

## Slide 4
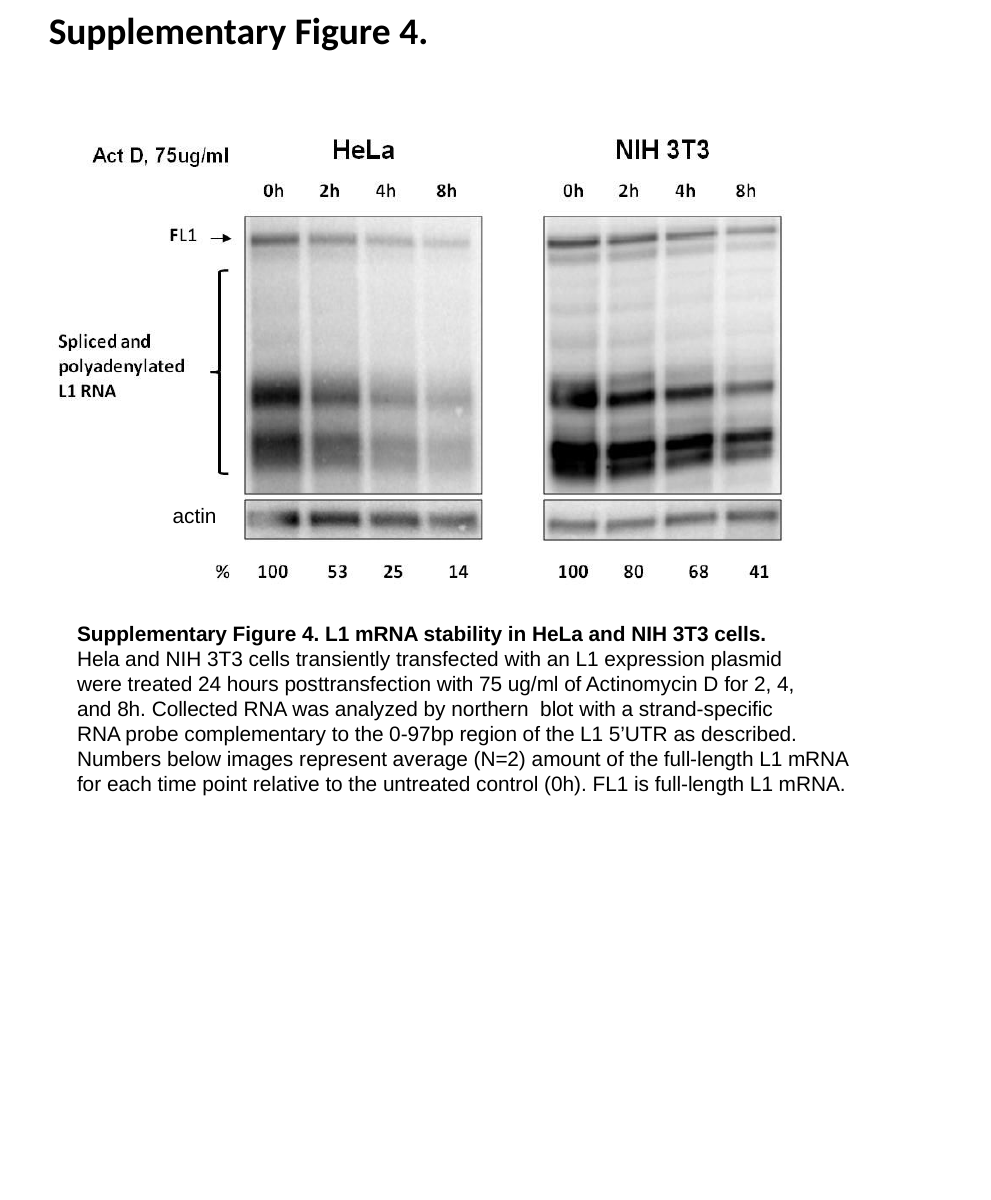

Supplementary Figure 4.
actin
Supplementary Figure 4. L1 mRNA stability in HeLa and NIH 3T3 cells.
Hela and NIH 3T3 cells transiently transfected with an L1 expression plasmid
were treated 24 hours posttransfection with 75 ug/ml of Actinomycin D for 2, 4,
and 8h. Collected RNA was analyzed by northern blot with a strand-specific
RNA probe complementary to the 0-97bp region of the L1 5’UTR as described.
Numbers below images represent average (N=2) amount of the full-length L1 mRNA
for each time point relative to the untreated control (0h). FL1 is full-length L1 mRNA.

## Slide 5
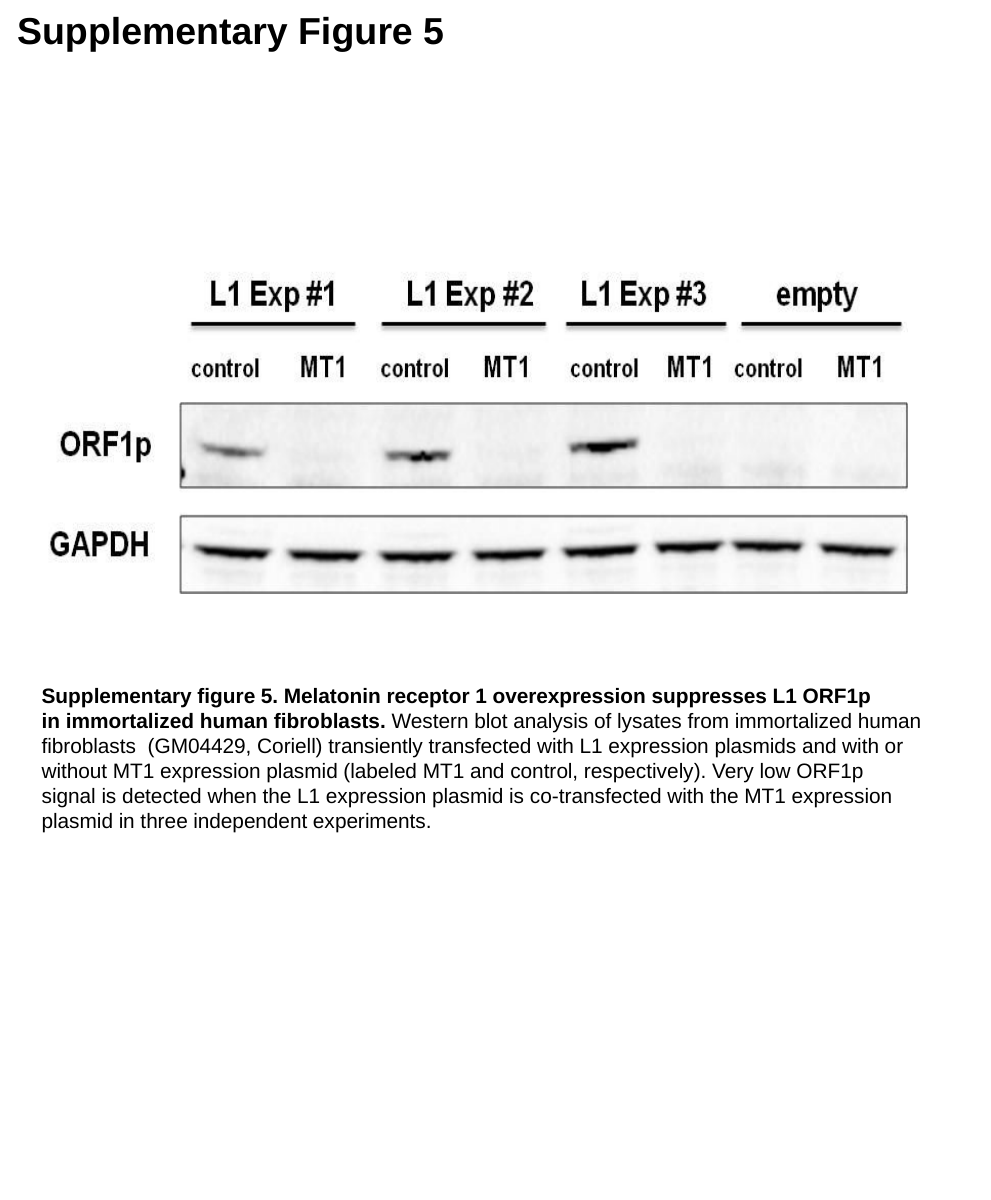

Supplementary Figure 5
Supplementary figure 5. Melatonin receptor 1 overexpression suppresses L1 ORF1p
in immortalized human fibroblasts. Western blot analysis of lysates from immortalized human
fibroblasts (GM04429, Coriell) transiently transfected with L1 expression plasmids and with or
without MT1 expression plasmid (labeled MT1 and control, respectively). Very low ORF1p
signal is detected when the L1 expression plasmid is co-transfected with the MT1 expression
plasmid in three independent experiments.

## Slide 6
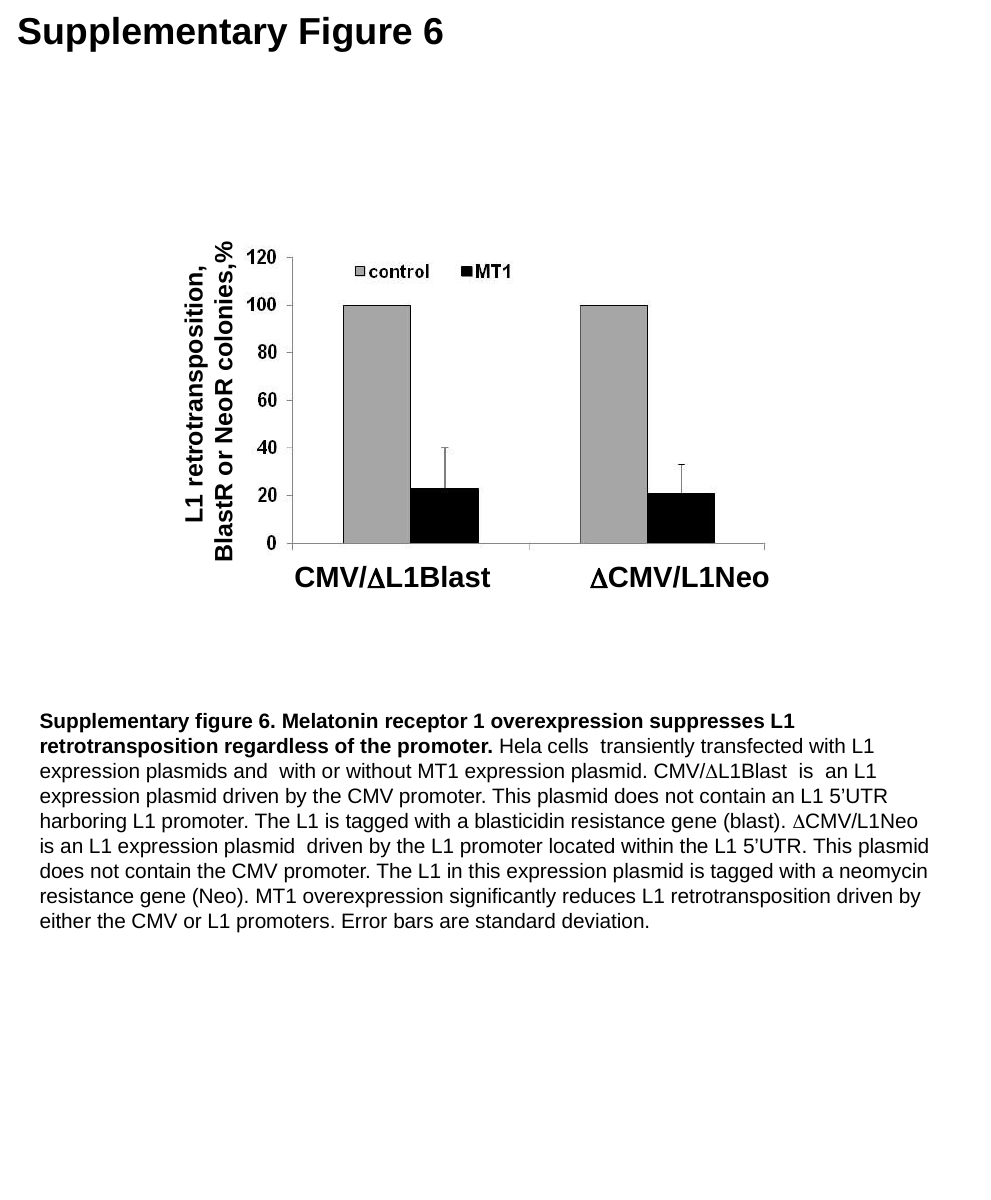

Supplementary Figure 6
 L1 retrotransposition,
BlastR or NeoR colonies,%
 CMV/DL1Blast DCMV/L1Neo
Supplementary figure 6. Melatonin receptor 1 overexpression suppresses L1
retrotransposition regardless of the promoter. Hela cells transiently transfected with L1
expression plasmids and with or without MT1 expression plasmid. CMV/DL1Blast is an L1
expression plasmid driven by the CMV promoter. This plasmid does not contain an L1 5’UTR
harboring L1 promoter. The L1 is tagged with a blasticidin resistance gene (blast). DCMV/L1Neo
is an L1 expression plasmid driven by the L1 promoter located within the L1 5’UTR. This plasmid
does not contain the CMV promoter. The L1 in this expression plasmid is tagged with a neomycin
resistance gene (Neo). MT1 overexpression significantly reduces L1 retrotransposition driven by
either the CMV or L1 promoters. Error bars are standard deviation.

## Slide 7
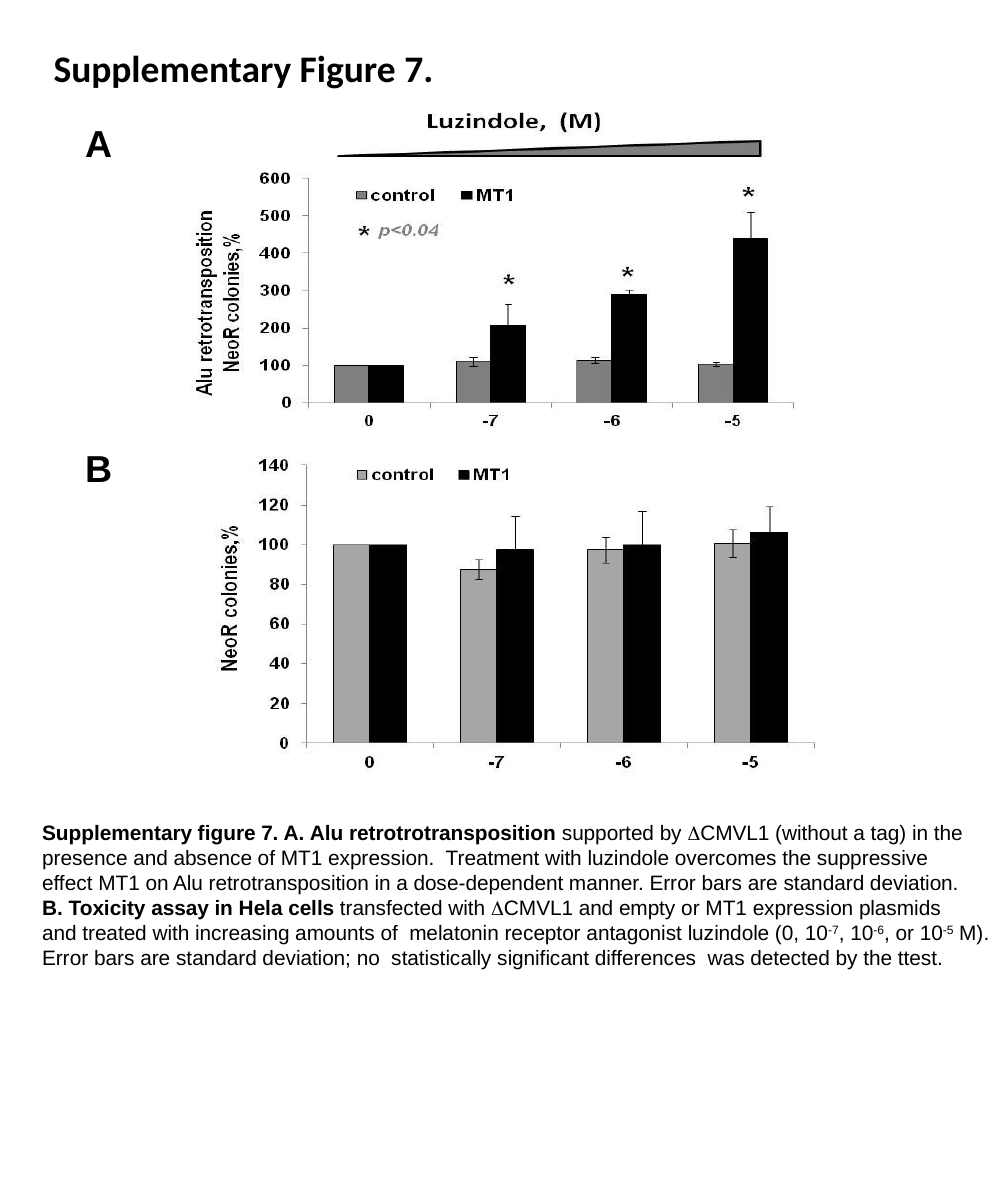

Supplementary Figure 7.
A
B
Supplementary figure 7. A. Alu retrotrotransposition supported by DCMVL1 (without a tag) in the
presence and absence of MT1 expression. Treatment with luzindole overcomes the suppressive
effect MT1 on Alu retrotransposition in a dose-dependent manner. Error bars are standard deviation.
B. Toxicity assay in Hela cells transfected with DCMVL1 and empty or MT1 expression plasmids
and treated with increasing amounts of melatonin receptor antagonist luzindole (0, 10-7, 10-6, or 10-5 M).
Error bars are standard deviation; no statistically significant differences was detected by the ttest.

## Slide 8
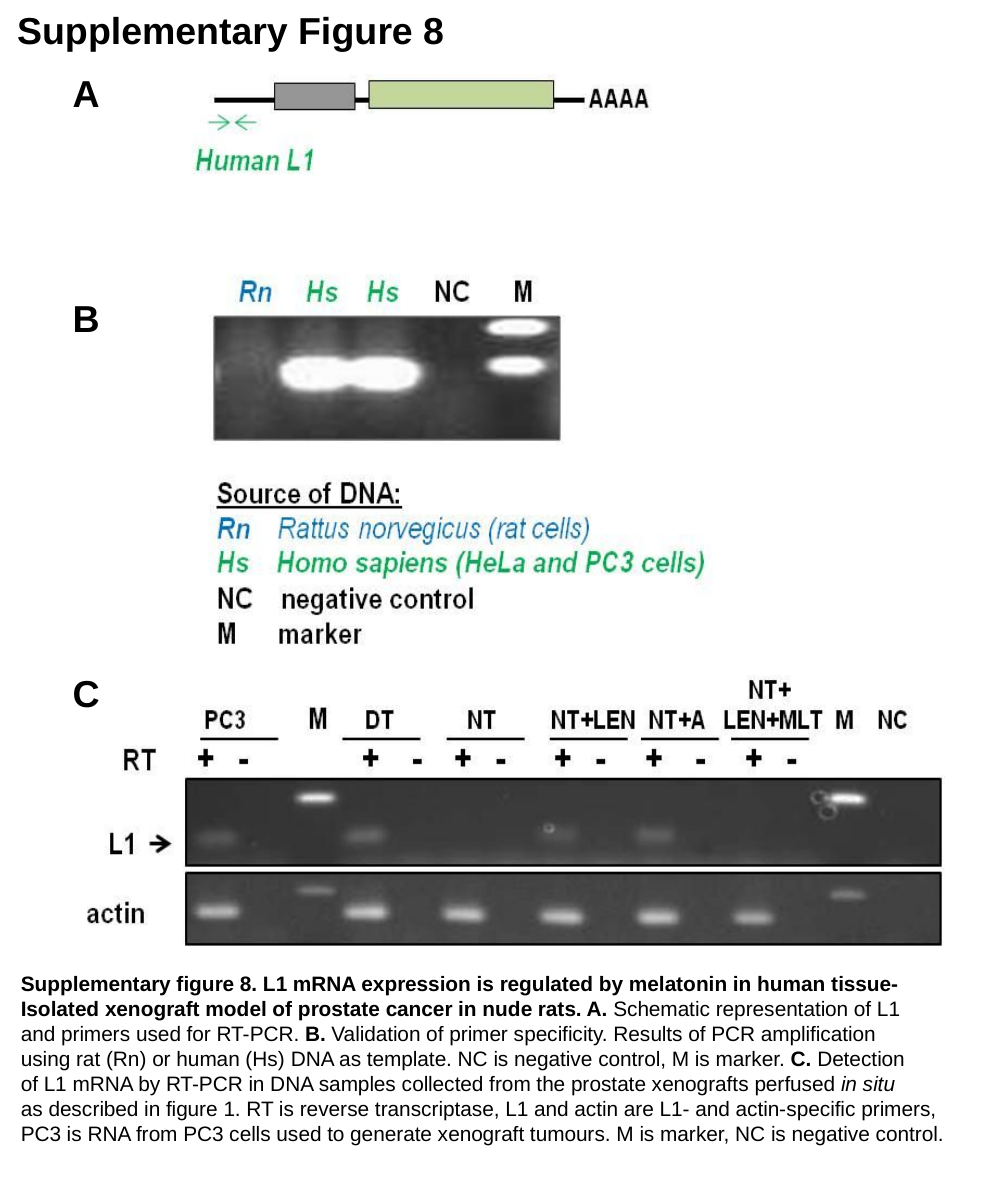

Supplementary Figure 8
A
B
C
Supplementary figure 8. L1 mRNA expression is regulated by melatonin in human tissue-
Isolated xenograft model of prostate cancer in nude rats. A. Schematic representation of L1
and primers used for RT-PCR. B. Validation of primer specificity. Results of PCR amplification
using rat (Rn) or human (Hs) DNA as template. NC is negative control, M is marker. C. Detection
of L1 mRNA by RT-PCR in DNA samples collected from the prostate xenografts perfused in situ
as described in figure 1. RT is reverse transcriptase, L1 and actin are L1- and actin-specific primers,
PC3 is RNA from PC3 cells used to generate xenograft tumours. M is marker, NC is negative control.
